# In vitro model of production of antibodies*; a* new approach to reveal the presence of key bacteria in polymicrobial environments

**DOI:** 10.1186/s12866-016-0821-5

**Published:** 2016-09-09

**Authors:** Chongcong Wu, Sravya Nakka, Sepahdar Mansouri, Torbjörn Bengtsson, Tayeb Nayeri, Fariba Nayeri

**Affiliations:** 1 The Institute of Protein Environment Affinity Surveys (PEAS Institut), Linköping, Sweden; 2 Department of Medical Sciences, Örebro University, Örebro, Sweden; 3 Maternal and Children Health Care Hospital of Zhuhai City, Zhuhai, China; 4 Division of Infectious Diseases, Department of Medical and Health Sciences, Linköping University, Linköping, Sweden

**Keywords:** Enterococcus, Biofilms, Antibodies, Detection, Electron microscopy, Lymphocytes, Surface plasmon resonance

## Abstract

**Background:**

There is a rapid emergence of multiple resistant gram-negative bacteria due to overuse of antibiotics in the treatment of infections. Biofilms consist of polymicrobial communities that survive the host’s defense system. The key bacteria in biofilms are slow growing and support an attachment and rapid growth of other microorganisms. Current antimicrobial strategies often fail due to poor diagnosis of key pathogens in biofilms.

The study aims to develop anti-bacterial human antibodies in vitro from patients who had recently undergone a systemic infection by pathogenic bacteria and to use these antibodies as a tool for detecting bacteria in biofilms.

**Methods:**

Lymphocytes were separated from whole blood of patients (*n* = 10) and stimulated with heat-killed bacteria to produce antibodies in vitro. The specificity of antibodies in recognizing the bacteria against which they were directed was evaluated by surface plasmon resonance system (SPR) and electron microscopy. The ulcer secretions from patients with chronic and acute leg ulcers and healthy controls were analyzed by the SPR system and the results were compared with culture studies.

**Results:**

The produced antibodies recognized bacteria with high sensitivity (SPR). The antibodies against *Enterococcus fecalis* bound specifically to the microorganism in a bacterial co-culture that was visualized by electron microscopy.

**Conclusion:**

In the present work, a method for producing specific antibodies against bacteria is introduced to recognize bacterial components in body fluids of patients suffering from pathogenic biofilms. This diagnostic technique may be most useful in clinical microbiology and in the choice of antibiotics in the treatment of serious infections.

**Electronic supplementary material:**

The online version of this article (doi:10.1186/s12866-016-0821-5) contains supplementary material, which is available to authorized users.

## Background

**Fig. 1:**
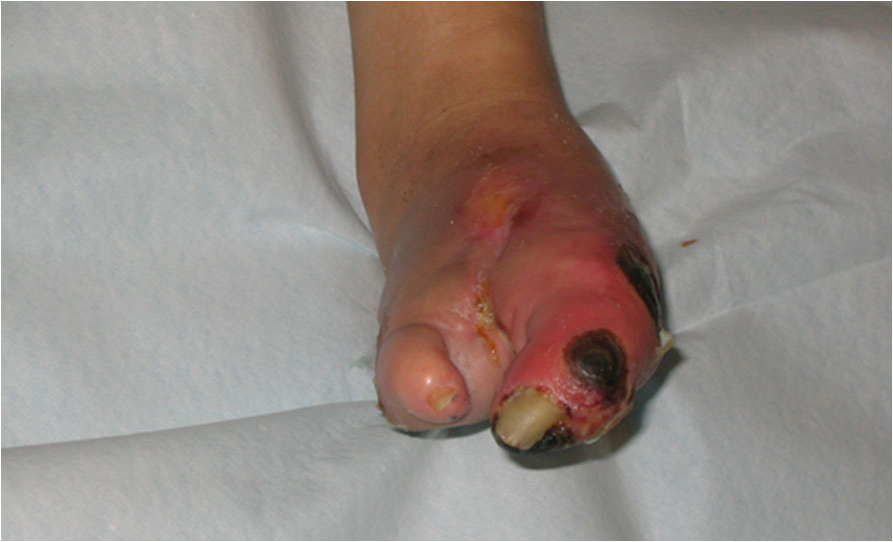
Chronic ulcer in a patient with persistent *Enterococcus fecalis* infection and organ dysfunction in 2006

In late autumn 2008, a 49-year old woman died. She suffered from type 1 diabetes, familial mediterranean fever, peripheral neuropathy, and foot ulcers. In February 2008, she was admitted to the Department of Infectious Diseases, University Hospital in Linköping for amputation of her left foot and received piperacillin-tazobactam intravenously and a blood transfusion post-operatively (Fig. 1). Despite this, her clinical status deteriorated critically within one week and she developed acute liver and renal insufficiency. The suggested diagnosis was hemolytic uremic syndrome and the result of an allergic reaction to piperacillin-tazobactam. She was treated with plasmapheresis, corticosteroids, imipenem, and metronidazole. The culture from the foot ulcer prior to amputation revealed growth of *Enterococcus fecalis* (*E. fecalis*) together with several gram-negative bacterial strains. She recovered partially, but the renal function was permanently destroyed and she was treated with hemodialysis and released on March 11^th^ 2008. On March 24^th^, she was readmitted to the Department of Infectious Diseases for fever, shivering, and diarrhea. The blood cultures revealed growth of *E. fecalis* and *Staphylococcus aureus*. She was transferred to the Department of Cardiology, University Hospital in Linköping for mitral valve endocarditis and underwent surgery for biological valve prosthesis. The antibiotic treatment was composed of meropenem, vancomycin, and clindamycin and continued for 8 weeks. The heart valve culture revealed *E. fecalis*. She was dismissed on June 5^th^ 2008 with moxifloxacin. She developed vomiting after 5 days at home and was readmitted on June 23^rd^. Blood cultures revealed growth of *E. fecalis* again and she was treated with vancomycin. Echocardiography showed a paravalvular leak that was treated at the Department of Cardiology. On July 28^th^ 2008, while still on the ward, she experienced sudden temporary blindness. A computed tomography (CT) scan did not show new pathologic signs that might explain the symptom. Doktacillin was complementary to vancomycin in the treatment. However, she developed hemolytic anemia and antibiotic therapy was interrupted on August 8^th^. She suffered from anxiety, depression, severe heart failure, pain, and loss of vision, but she had no fever. Blood tests revealed a high sedimentation rate, anemia, high white blood cell count, and a high level of C-reactive protein. Blood cultures from eight different occasions yielded no growth. Two short episodes of antibiotic therapy with tigecycline and imipenem were interrupted due to negative blood cultures. The symptoms were judged as Mediterranean fever and she was transferred to the Department of Nephrology with high dose of corticosteroids on October 1^st^. She died October 13^th^ due to septic shock and *E. fecalis* grew in all blood cultures that were taken before she died.

Persistent infection in biofilms has been the subject of clinical studies since 1981. Biofilms are a collection of microbes that adhere to surfaces by producing a matrix that shields them from environmental elements. It has been speculated that bacteria colonizing chronic wounds are part of the highly persistent biofilms [1]. Molecular analyses of chronic wound specimens revealed diverse polymicrobial communities but it has been very difficult to identify specific bacteria of the entire individual, especially strictly anaerobic bacteria, by culture methods [2]. Traditional culturing methods may be extremely biased as a diagnostic tool as they select for easily cultured organisms e.g. *S. aureus,* but not bacteria difficult to culture such as anaerobes [3]. The formation of bacterial biofilms could lead to chronic inflammation. Detachment of the biofilm enables bacteria to enter into the blood stream, causing bacteremia and vascular embolism [4]. The establishment of a non-cultural method for analysis of infections may help to identify the key bacteria that cause pathogenic biofilms [5–7]. Thus, it is most important to identify the underlying causative infectious agent in the symbiotic community of biofilms in order to antagonize the development of a suitable environment for opportunistic pathogens and thereby improve the wound healing process.

*E. fecalis* is a successful pathogen and is able to adapt to the hostile environment to grow within the host and has been shown to facilitate the survival of other bacteria within the biofilms [8]. Therapy directed to eliminate adherent gram-positive bacteria, such as *E. fecalis*, may effectively destroy the biofilm by disturbing the growth of rapid growing gram-negative bacteria [9–11].

The stimulation of memory cells from an isolated and cultured lymphocyte population with antigens similar to the subsequent infection could simplify the process of antigen specific IgG secretion [12].

SPR is a real-time optical technique that can simultaneously determine the affinity of a protein for several ligands by using individual surfaces on a sensor chip [13].

As part of serial studies to identify the role of the key bacteria in several associated diseases, this study aims to:

- Develop anti-bacterial human antibodies in vitro from patients who have been treated for systemic infection.
- Immobilize the produced antibodies in a SPR system and use the response to injected heat-killed pathogens for assessment of specificity.
- Analyze the ulcer secretions from patients and healthy volunteers by the SPR system and compare the results to data from culture methods.
- To use these antibodies as an alternative tool for detecting bacteria in biofilms.

## Methods

### Study subjects

The blood samples were collected at convalescence from 10 patients (described in the following section) who met the study leader at Department of Infectious Diseases and attended a follow-up visit within 4–8 weeks after onset of systemic infection. The informed consent was obtained and the samples were handled unidentified.

The ulcer secretion was collected from subjects (*n* = 24, described in the following section). This material has been presented previously [14]. The study was approved by the local ethics committee at Linköping University Hospital. All participants provided written consent (03–322, M133-09).

### Blood samples

Blood samples were gathered in EDTA tubes (Venoject K2K, Terumo Europe NV, Leuven, Belgium) from patients who had systemic infections caused by *E. fecalis* (male, 70 years old, septicemia)*, S. aureus* (male, 26 years old, severe periodontitis, ulcers and septicemia), *Pseudomonas aeruginosa* (*p. aeruginosa*) (female, 35 years old, decubital ulcer), *Staphylococcus epidermidis* (*S. epidermidis*) (female, 76 years old, prosthesis infection), *Clostridium perfringens* (female, 75 years old, decubital abscess), *Bacterioides fragilis* (male, 61 years old, perianal abscess and septicemia), *Prevotella oralis* (male, 60 years old, severe periodontitis and bacteremia), *Escherichia Coli* (*E. coli*) (female, 38 years old, pyelonephritis) and *Streptococcus sp.* (78 years old male, septicemia) at convalescence.

### Ulcer secretion

Ulcer secretion was gathered from ten patients (8 women, 44–89 years of age, median 76 years old) with chronic leg ulcers that had been stable for at least 6 months. The etiologies of the ulcers were venous insufficiency or a combined venous and mild arterial insufficiency determined by clinical judgment and physiological measurements, i.e., toe and ankle pressure measurements. Cultures from the ulcers revealed growth of several species of bacteria; *S. aureus*, *Enterobacter cloacae*, *P. aeruginosa*, *E. fecalis,* and *Proteus mirabilis*. Skin biopsies were taken from the left arm of nine healthy volunteers (all women, 40–60 years of age, median: 54 years) and were used as controls. The ulcer secretion was collected within 24 h after injury and the cultures from these healthy controls were negative. Ulcer secretions were also collected from 5 patients within 2 weeks of operation for breast cancer without any signs of metastasis (all women, 31–85 years of age, median 64 years). Cultures revealed growth of *S. aureus* in two cases and were negative in the rest of these patients.

### Collection of ulcer secretion

During a 24-h period, equal amounts of ulcer secretion were collected by absorption using 1 cm^2^ of absorbent material (Mepilex, Mölnly Health Care AB, Box 13080, SE 40252, Göteborg, Sweden) placed under the dressing and then transferred into a flask (scintillation vial 20 mL, Sarstedt AB, SE- 26151 Landskrona, Sweden) containing 5 ml physiological sodium chloride (NaCl). The sample was vortexed (Vortex-Genie, Scientific Industries, Inc., New York, USA) and centrifuged at 3000 × g for 10 min. The supernatant was transferred into tubes (Nunc Cryo Tube, Nunc Brand Products, Denmark) and stored at −70 °C before analysis.

### In vitro antibody production

Whole blood was diluted in 0.9 % NaCl solution in a 1:1 ratio and lymphocytes were isolated by density gradient centrifugation using lymphoprep solution (Axis-shield PoC, Oslo, Norway) upon centrifugation at 1800 × *g* at 20 °C for 20 min. A clear distinct layer of mononuclear cells containing lymphocytes was carefully pipetted from the tube and cultured in L-15 medium (ATCC, Borås, Sweden), supplemented with 10 % fetal bovine serum (FBS; Sigma Aldrich, Stockholm, Sweden), at 37 °C. The lymphocytes were then stimulated with heat-killed *E. fecalis* (CCUG, Gothenberg University, Sweden) at an optical density (OD) of 1 and incubated at 37 °C for 3 weeks (Additional file 1). After 3 weeks, the medium was collected and centrifuged at 3000 × g for 10 min to pellet the cells. The supernatant was filtered using 0.45 μm sterile filters (Thermofisher Scientific, Stockholm, Sweden) and centrifuged again using 100 KDa Amicon Microcon Centrifugal Filters (Millipore, Molsheim, France) at 3000 × g for 60 min. Antibodies with molecular weight over 100KDa were recovered from the filters by pipetting and were stored at −70 °C until further use.

### Electron microscopy examination of bacteria

A bacterial co-culture of *E. fecalis*, *S. aureus* and *E. coli* was prepared by diluting the heat-killed bacteria in 1:100 ratio in PBS (pH 7.4, Apoteket, Linköping, Sweden), and in vitro specific anti-*E. fecalis* antibodies were diluted 1:200 in PBS. The bacteria were incubated overnight with anti- *E. fecalis* antibodies at 4 °C. Samples were then incubated with nano gold-anti-human IgG antibodies (Nanoprobes, New York, USA) diluted 1:10 in PBS for 2 h. The specimen was mounted on the formvar coated copper grids and counter stained with 2 % Osmium tetroxide (Sigma-Aldrich, Sweden) and were analyzed using TEM1230 Gantan Electron microscope (Linköping University, Sweden).

### Analysis of ligand-binding affinity using surface plasmon resonance

The SPR measurements and ligand immobilization procedures were conducted at 760 nm in a fully automatic Biacore 2000 instrument (GE-Healthcare GmbH, Uppsala, Sweden) equipped with four flow cells, and the temperature was 25 °C in all experiments. The running buffer was HBS-EP (0.01 M HEPES, 0.15 M NaCl, 3 mM EDTA, 0.005 % surfactant P20, pH 7.4) (GE-Healthcare GmbH). Ligands were coupled to carboxymethylated dextran CM5 chips (GE Healthcare GmbH) by conventional carbodiimide chemistry using 200 mM N-ethyl-N-(3 diethylaminopropyl) carbodiimide (EDC) and 50 mM N-hydroxysuccinimide (NHS). The activation time was 7 min, followed by a 7 min ligand injection. Deactivation of the remaining active esters was performed by a 7 min injection of ethanolamine/hydrochloride, pH 8.5. A flow rate of 5 μl/min was used during the immobilization and measurement procedures. Antibodies developed against bacteria were diluted 1:10 in 10 mM acetate buffer, pH 4.5, below the isoelectric point of the protein, thus enhancing the electrostatic interactions between the dextran matrix and the ligands. Anti-guinea pig IgG and anti-human IgG (Sigma Aldrich) and heat-killed ATCC bacterial strains (CCUG, Gothenburg, Sweden) were used as controls. The contact time was 7 min, which resulted in immobilization levels between 15000 and 40000 response units (RU). Ulcer secretions were diluted 1:1 in PBS (Apoteket AB, Umeå, Sweden). Injection of equal mixture of 1 M NaCl and 10 mM glycine, pH 2, followed by one injection of borate pH 8.5, were used for regeneration (1 min).

### Statistics

Test performance of antibodies immobilized in SPR system in identification of bacteria in ulcer secretions, compared to culture results, were calculated using the following formulas:

$$ \mathrm{Negative}\ \mathrm{P}\mathrm{redictive}\ \mathrm{value}\ \left(\mathrm{N}\mathrm{P}\mathrm{V}\right)=\frac{\mathrm{Number}\ \mathrm{of}\ \mathrm{true}\ \mathrm{negatives}}{\left(\mathrm{N}\mathrm{umber}\ \mathrm{of}\ \mathrm{true}\ \mathrm{negatives}\right) + \left(\mathrm{N}\mathrm{umber}\ \mathrm{of}\ \mathrm{false}\ \mathrm{negatives}\right)} $$

$$ \mathrm{Positive}\ \mathrm{P}\mathrm{redictive}\ \mathrm{value}\ \left(\mathrm{P}\mathrm{P}\mathrm{V}\right) = \frac{\mathrm{Number}\ \mathrm{of}\ \mathrm{true}\ \mathrm{positives}}{\left(\mathrm{Number}\ \mathrm{of}\ \mathrm{true}\ \mathrm{positives}\right) + \left(\mathrm{Number}\ \mathrm{of}\ \mathrm{false}\ \mathrm{positives}\right)} $$

$$ \mathrm{Specificity} = \frac{\mathrm{Number}\ \mathrm{of}\ \mathrm{true}\ \mathrm{negatives}}{\left(\mathrm{Number}\ \mathrm{of}\ \mathrm{true}\ \mathrm{negatives}\right) + \left(\mathrm{Number}\ \mathrm{of}\ \mathrm{false}\ \mathrm{positives}\right)} $$

$$ \mathrm{Sensitivity} = \frac{\mathrm{Number}\ \mathrm{of}\ \mathrm{true}\ \mathrm{positives}}{\mathrm{Number}\ \mathrm{of}\ \mathrm{true}\ \mathrm{positives} + \left(\mathrm{Number}\ \mathrm{of}\ \mathrm{false}\ \mathrm{negatives}\right)} $$

## Results

The specificity of the developed antibodies was studied by the assessment of binding affinity of heat-killed bacterial strains to antibodies immobilized in the SPR system (Table 1). The SPR analysis of ulcer secretions using biosensor chips immobilized with antibodies developed against *P. aeruginosa*, *S. aureus*, *E. fecalis*, and *S. epidermidis* determined that *S. aureus* was found in 6/10 cultures from chronic ulcers and there was binding affinity to antibodies against *S. aureus* in 5/6 SPR analyses. *S. aureus* grew in 2/5 ulcer secretions from patients that had undergone breast cancer surgery and there was a binding affinity to *S. aureus* antibodies in both cases. *E. fecalis* grew in 1/10 ulcer secretion from chronic ulcers, however there was a positive binding to antibodies against *E. fecalis* in 3/10 specimens. There was no binding affinity to antibodies against *E. fecalis* in the ulcer secretions from healthy staff or patients who underwent breast cancer surgery (Table 2).

**Table 1:** Specificity test of in vitro antibodies

Controls	Anti-*S. aureus*	Anti-*S. epidermidis*	Anti-E. fecalis	Anti-*P. aeruginosa*	Anti-*C. perfringens*	Anti- *B. fragilis*	Anti-*P. oralis*	Anti*-E. coli*	*Anti-Streptococcus sp.*
*S. aureus*	250	0	0	0	0	0	0	0	0
*S. epidermidis*	0	103	0	0	0	0	0	0	0
*E. fecalis*	0	0	244	0	0	0	0	0	0
*P. aeruginosa*	0	0	0	36.4	0	0	0	0	0
*C.perfringens*	0	0	0	0	31.8	0	0	0	0
*B. fragilis*	0	0	0	0	0	230.1	0	0	0
*P. oralis*	0	0	0	0	0	0	570	0	0
*E. coli*	0	0	0	0	0	0	0	185.5	0
*Streptococcus sp.*	0	0	0	0	0	0	0	0	278.3
Anti-guinea pig IgG	0	0	0	0	0	0	0	0	0
Anti-human IgG	279	140	646.5	439	342.3	75.8	809.2	234.6	461.1

**Table 2:** Affinity of ulcer secretions to the immobilised antibodies in SPR system and the correlation to culture results

Ulcer secretion	Culture results	Binding to the produced antibodies in SPR system			
		Anti-*P. aeruginosa*	Anti-*S. aureus*	Anti-*E. fecalis*	Anti-*S. epidermidis*
Ulcer secretion from patients with chronic ulcer					
1	*Staphylococcus aureus + Enterobacter cloacae*	negative	positive	negative	positive
2	*Staphylococcus aureus + Echerichia coli*	negative	positive	negative	negative
3	*Staphylococcus aureus + Pseudomonas aeroginosa*	negative	positive	negative	negative
4	*Staphylococcus aureus*	negative	negative	negative	negative
5	*Proteus mirabilis + coagulase negative staphylococcus*	negative	negative	positive	positive
6	negative	negative	negative	negative	negative
7	*Pseudomonas aeruginosa + Enterococcus fecalis*	positive	negative	positive	negative
8	*Staphylococcus aureus*	negative	positive	negative	positive
9	*Staphylococcus aureus*	negative	positive	positive	positive
10	negative	negative	negative	negative	negative
Ulcer secretion from patients post breast cancer surgery					
1	negative	negative	negative	negative	positive
2	negative	negative	negative	negative	positive
3	negative	negative	negative	negative	positive
4	*Staphylococcus aureus*	negative	positive	negative	negative
5	*Staphylococcus aureus*	negative	positive	negative	negative
Ulcer secretion from healthy volunteers post skin biopsy in fore-arm					
1	negative	negative	negative	negative	positive
2	negative	negative	negative	negative	negative
3	negative	negative	negative	negative	negative
4	negative	negative	negative	negative	positive
5	negative	negative	negative	negative	positive
6	negative	negative	negative	negative	positive
7	negative	negative	negative	negative	positive
8	negative	negative	negative	negative	positive
9	negative	negative	negative	negative	positive

There was no growth of bacteria in cultures of ulcer secretions from the forearm in the healthy staff post biopsy. However, there was a positive binding to antibodies against *S. epidermidis* in 7/9 specimens.

Test performance for detection of pathogens in ulcer secretions by SPR using in vitro antibodies, compared with conventional culture techniques, showed results for *S. aureus* with 87.5 % sensitivity, 100 % specificity, positive predictive value (PPV) 100 % and negative predictive value (NPV) 94 % and for *E. fecalis* with 100 % sensitivity, 91.3 % specificity, PPV 33.3 % and NPV 100 % (Tables 2 and 3).

**Table 3:** Test performance of binding to the produced antibodies immobilized in SPR system in comparison to conventional ulcer secretion cultures

SPR/culture	False positive (SPR)	True positive (culture)	False negative (SPR)	True negative (culture)	Total
*Staphylococcus aureus*	0	7	1	16	24
*Enterococcus fecalis*	2	1	0	21	24

Sensitivity for *Staphylococcus aureus* was 87.5 %, specificity 100 %, PPV 100 % and NPV 94 %. Sensitivity for *Enterococcus fecalis* was 100 %, specificity 91.3 %, PPV 33.3 % and NPV 100 %

**Fig. 2:**
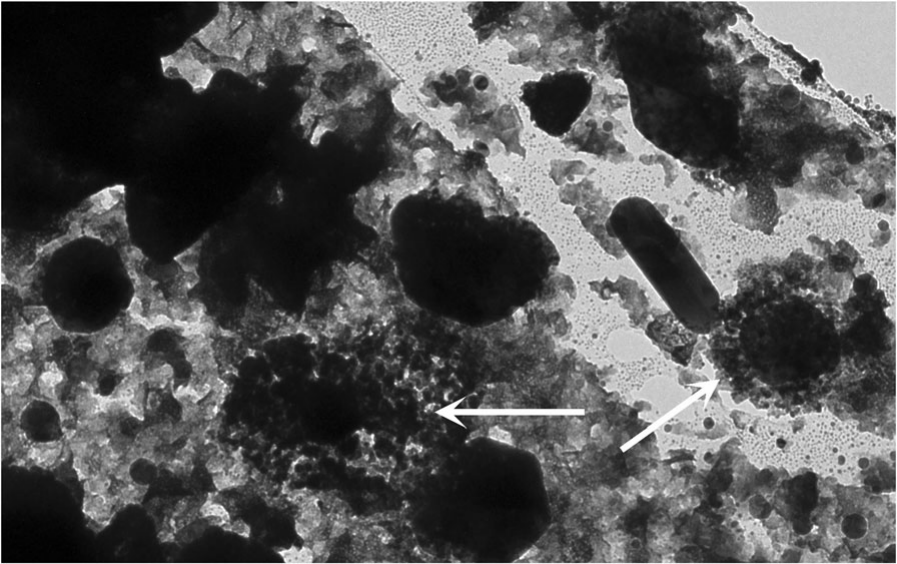
Co-culture of three bacterial strains: *Staphylococcus aureus*, *Enterococcus fecalis*, and *Escherichia coli*. Anti- *Enterococcus fecalis* antibodies incubated with the co-culture bound to some cocci but not to the other cocci or rods (white arrows). This interaction was visualized by electron microscopy by adding gold labled anti human IgG antibodies to the medium

**Fig. 3:**
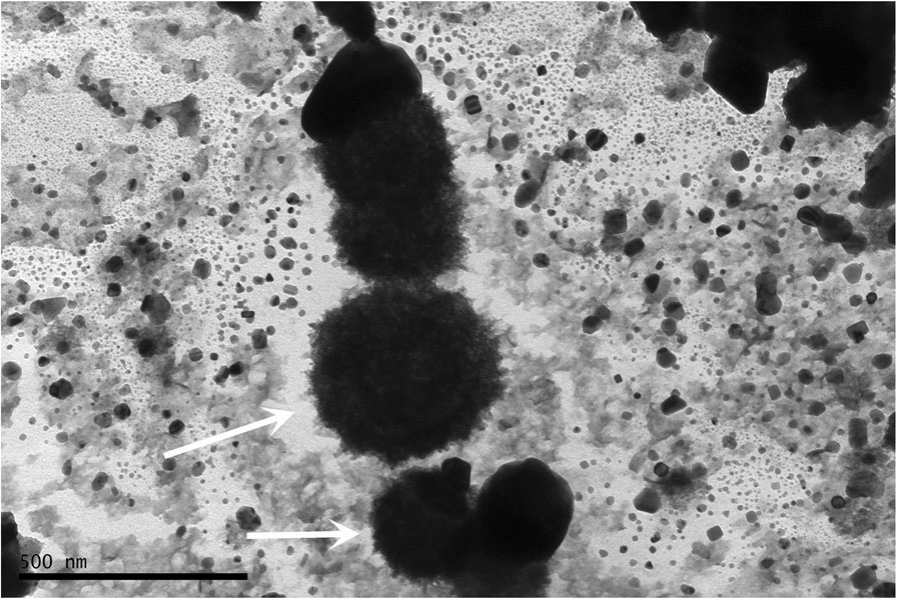
Co-culture of *Enterococcus fecalis* and *Staphylococcus aureus* incubated with anti-*Enterococcus fecalis* antibodies and visualized by electron microscopy. Please note the antibodies covering some cocci in the co-culture of two bacterial strains (white arrow)

The electron microscope images showed a specific interaction of anti-*E. fecalis* antibodies to *E. fecalis* in a monoculture preparation but not in a monoculture preparations of *S. aureus* or *E. coli* (Figs. 2 and 3). Furthermore, in a bacterial co-culture preparation containing the three bacterial strains (*E. fecalis*, *S. aureus*, and *E. coli*), anti-*E. fecalis* antibodies bound specifically only to the surface of few cocci (shown in arrows) but not to the other bacteria (Additional file 2).

## Discussion

In the present work, the case of a middle-aged woman with persistent infection caused by *E. fecalis* and the ultimate outcome motivated a development of a non-culture bacterial detection method in management of complicated patient cases. The objective was to further increase the knowledge regarding the detection of life-threatening silent infections. Anti-bacterial in vitro human antibodies were developed from patients at convalescence and immobilized in a SPR system and used for assessment of presence of bacteria in ulcer secretions. Compared to culture results, the produced antibodies could recognize the microorganism with quite high test performance.

In the field of medical diagnostics and the treatment of patients, the practitioner only has a narrow perspective on the factual preconditions of disease development and the true origin of the symptoms. Diagnostic tools give the clinician valuable insight into the characteristics of the pathology and help in choosing the best path of intervention [15]. Previous medical records are seldom studied in detail and it is quite acceptable for medical personnel to attempt to treat the complications of chronic inflammatory processes by managing the symptoms and organ failures temporarily [16]. However, a great number of different infectious agents may contribute to the patient’s sicknesses and sometimes the main cause is identified and successfully treated [17–20]. Yet, the reasons for and circumstances in which there is a transition from acute to chronic infection and inflammation are still not well understood. To better understand this transition, the relevant factors and components have to be characterized and identified.

An acute inflammatory process is the immediate result of the body’s recognition of and reaction to a foreign pathogen and the body’s attempt to eliminate the intruder [21]. A prolonged chronic inflammatory process might depend on the persistence of the pathogens because of the nature of the invaders and/or the inability of the host to eliminate this intruder [22]. The constant host-microbe interactions are exhausting to the host body [23]. During an acute inflammatory response, the natural defensive reaction is more devastating to the body than the invasion itself. The contrary occurs during chronic inflammation, where the overgrowth of an otherwise hidden infection might cause septicemia, micro-circulatory defects, ischemia, and chronic organ failure [24]. Chronic sepsis and multiple organ failure can be consequences of a prolonged chronic inflammatory process [25]. In many cases, the source of inflammation is known, however with poor prospects of eliminating it. Bacteria in the planktonic shape might often be sensitive to immune responses and antibiotics, whereas the coexistence of bacteria in a biofilm makes them resistant to antibiotics. This also contributes to the development of new drug resistant strains [26, 27]. Therefore, it is most important to find out the reason why the infection succeeds in persisting, before it becomes invasive. As a model of chronic organ injury, we have investigated patients with chronic ulcers and have tried to clarify why some ulcers do not heal [28–31]. Periodontitis has been studied in our group with special focus on the role of *P. gingivalis* in chronic inflammatory diseases [32]. We have shown that stimulation of lymphocytes from a patient who suffered from periodontitis with *P. gingivalis* caused maturation of cells into antibody secreting, CD38-expressing plasma cells, 2–3 weeks after stimulation. The results from SPR analysis of crevicular fluid samples from patients with periodontitis and controls correlated significantly with RT-PCR results (*p* >0.05) [33].

Although the recent advances in science have succeeded in development of highly sensitive and specific nucleic-acid based methods, PCR analysis was not performed on the ulcer secretion that was assessed in the present study. The production of antibodies in vitro against bacteria was not meant to replace a highly technologic PCR method in detection of bacteria. We aimed to develop a method to specifically prove the presence of pathogenic bacteria. Upon detection of such bacteria we have recently succeeded to treat several complicated polymicrobial infections focused on gram-positive bacteria with antibiotic therapy including ampicillin or vancomycin [11]. This might have an impact on decreasing extended spectrum beta-lactamase (ESBL)**-**producing organisms that colonize chronic wounds. The finding that the SPR method did not detect culture verified bacteria in two ulcer secretions (Table 2), may be due to a low sensitivity of the SPR method. Though we have produced the antibodies at convalescence from lymphocytes of patients that had undergone infection with pathogenic bacteria, another reason for false negative result in two cases might be that the antibodies detect the pathogens with specified epitopes. This mechanism is one of our goals to be investigated in future studies.

## Conclusion

Biofilms are poorly investigated as a source of chronic inflammation and multi-resistant bacteria in biofilms and chronic organ dysfunction are huge clinical problems. The lack of scientific evidence demonstrating a near association between silent organ dysfunction and an infectious focus is a major problem in clinical medicine. We have attempted to produce antibodies using memory cells of patients that had undergone a severe infection caused by a pathogen in order to detect pathogens in polymicrobial environments. In future studies, we aim to identify and produce antibodies against epitopes that reflect the presence of pathogenic key bacteria in biofilms.

## Additional files

Additional file 1: Microscopic analysis of host lymphocytes after stimulation with heat killed *E. fecalis*. Lymphocytes cultured in L-15 medium with 10 % FBS were stimulated with the same bacteria which had previously infected the donor and immune response to pathogenic stimulation was observed at 40X resolution in light microscopy. Morphological changes were observed in activated lymphocytes. (JPG 375 kb) Additional file 2: Electron microscopic image of *Escherichia coli* incubated with Anti-BCG antibodies and gold labled anti human IgG secondary antibodies. (JPG 4702 kb)

## Abbreviations

*E.col*: *Escherichia coli*
*E.fecalis*: *Enterococcus fecalis*
EDS: N-ethyl-N-(3 diethylaminopropyl) carboiimide
ESBL: Extended spectrum beta-lactamase
Nacl: Natrium chloride
NHS: N-hydroxysuccinimide
NPV: Negative predictive value
*P. aeruginosa*: *Pseudomonas aeruginosa*
*P. gingivalis*: *Porphyromonas gingivalis*
PBS: Phosphate buffer solution
PPV: Positive predictive value
RU: Response unit
*S. aureus*: *Staphylococcus aureus*
*S. epidermidis*: *Staphylococcus epidermidis*
SPR: Surface plasmon resonance
