# Supplementary figures and images for: In vitro model of production of antibodies; a new approach to reveal the presence of key bacteria in polymicrobial environments
Source: BMC Microbiol. 2016 Sep 9;16(1):209. doi: 10.1186/s12866-016-0821-5 (PMC5017027; doi:10.1186/s12866-016-0821-5)

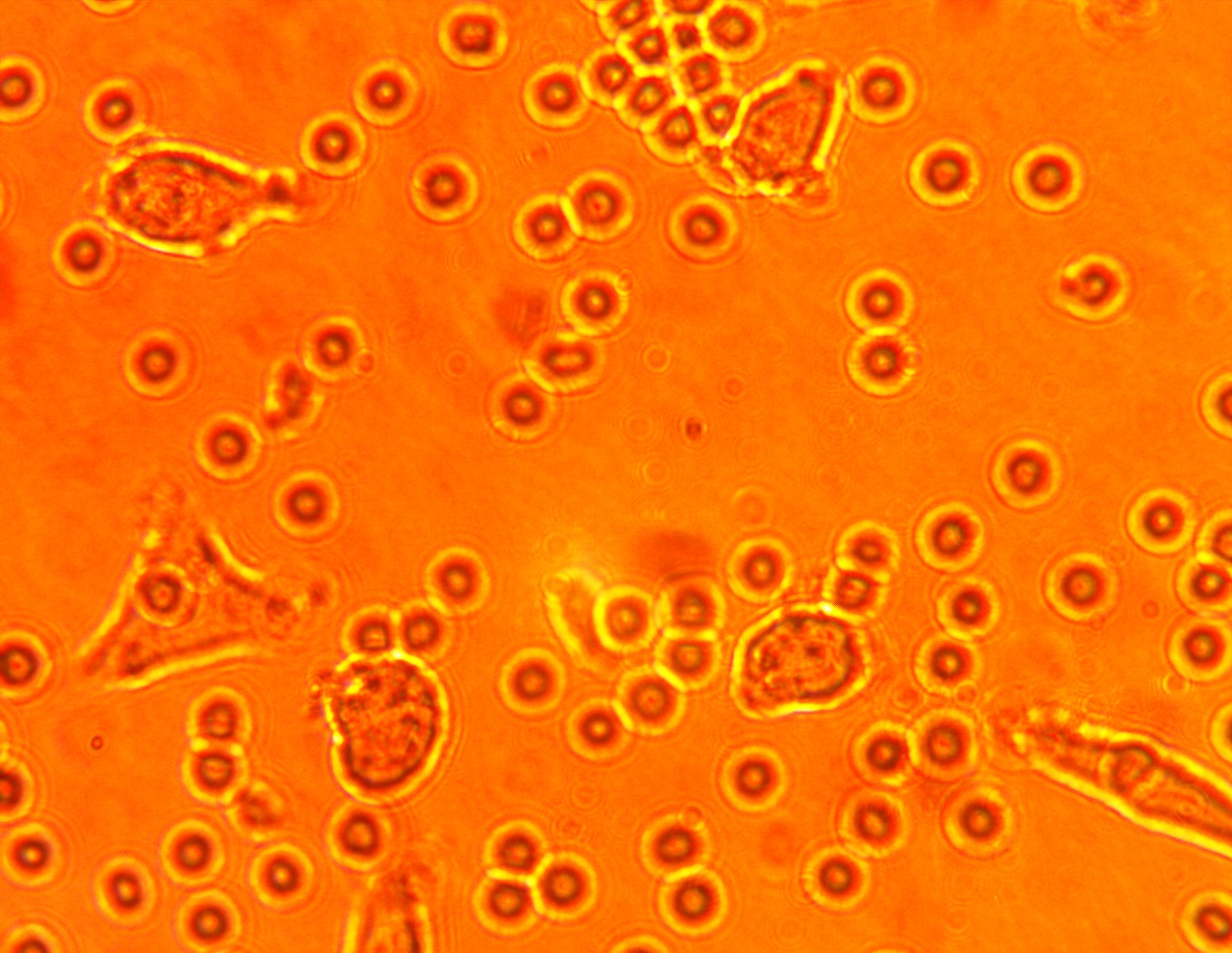

Supplement: Additional file 1: — Microscopic analysis of host lymphocytes after stimulation with heat killed E. fecalis. Lymphocytes cultured in L-15 medium with 10 % FBS were stimulated with the same bacteria which had previously infected the donor and immune response to pathogenic stimulation was observed at 40X resolution in light microscopy. Morphological changes were observed in activated lymphocytes. (JPG 375 kb) [file 12866_2016_821_MOESM1_ESM.jpg]

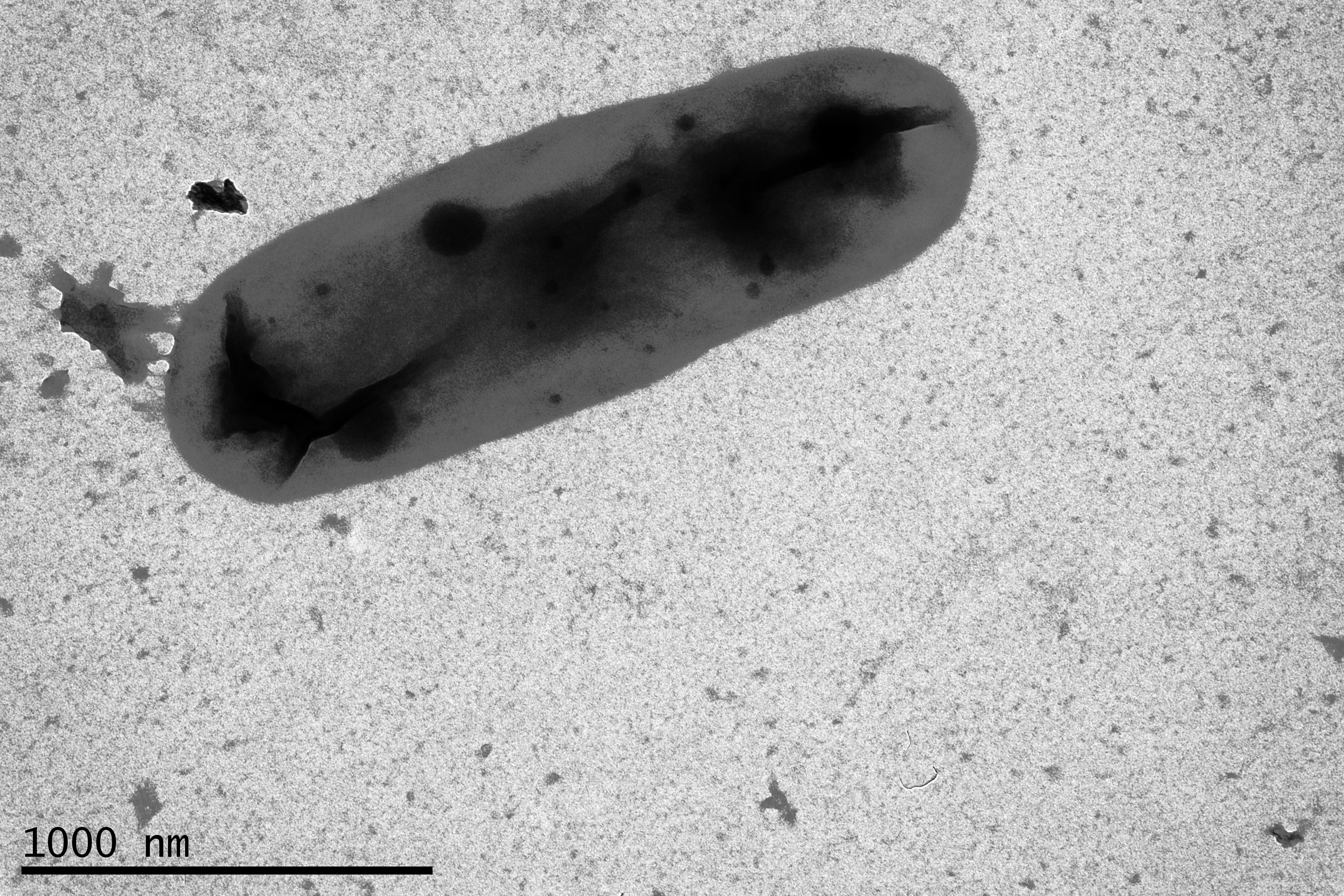

Supplement: Additional file 2: — Electron microscopic image of Escherichia coli incubated with Anti-BCG antibodies and gold labled anti human IgG secondary antibodies. (JPG 4702 kb) [file 12866_2016_821_MOESM2_ESM.jpg]
